# Influence of Process Parameters on the Tribological Behavior of PEO Coatings on CP-Titanium 4+ Alloys for Biomedical Applications

**DOI:** 10.3390/ma14185364

**Published:** 2021-09-17

**Authors:** Stephan Lederer, Serkan Arat, Wolfram Fuerbeth

**Affiliations:** DECHEMA Research Institute, 60486 Frankfurt am Main, Germany; arat@dechema.de (S.A.); wolfram.fuerbeth@dechema.de (W.F.)

**Keywords:** plasma electrolytic oxidation, micro-arc oxidation, titanium, wear resistance, corrosion resistance

## Abstract

Wear resistant ceramic coatings were generated on novel commercially pure titanium grade 4+ alloys by the plasma electrolytic oxidation technique (PEO) in an aluminate and zirconia containing electrolyte. The coatings were obtained adopting a full regular two-level factorial design of experiments (DoE) varying the PEO process parameters current density, repetition rate and duty cycle. The generated coatings were characterized with respect to its wear resistance and mechanical properties by reciprocal ball-on-flat tests and nanoindentation measurements. Thickness, morphology and phase formation of the PEO coatings was analyzed by scanning electron microscopy (SEM/EDS) and X-ray diffraction. XRD results indicate the formation of crystalline aluminium titanate (TiAl${}_{2}$O${}_{5}$) as well as t-ZrO${}_{2}$ and alumina leading to an increase in hardness and wear resistance of the PEO coatings. Evaluation of the DoE’s parameter interaction shows that the main effects for generating wear resistant coatings are current density and repetition rate. In particular, the formation of mechanically stable and adhesive corundum and zirconia containing coatings with increasing current density and frequency turned out to be responsible for the improvement of the tribological properties. Overall, the PEO processing significantly improves the wear resistance of the CP titanium base alloy.

## 1. Introduction

Titanium and its alloys have been used as common materials for biomedical implant technologies for several decades, due to their good mechanical properties, corrosion resistance and satisfying biocompatibility [1,2,3]. Currently, the titanium alloys established in the field of medical technologies are Ti Al6 V4 and Ti Al6 Nb7, which contain 6% aluminium and 4% vanadium or 7% niobium in addition to titanium, respectively [4]. However, titanium and its alloys suffer from poor wear resistance. In particular, titanium surfaces brought in contact with each other or with other metals tend to fretting already under light loads and little relative motion [5]. The interaction of wear and corrosion (tribocorrosion) may lead to a progressive degradation of the material [6]. Moreover, harmful metal ions such as aluminium or vanadium or even abraded nanoparticles can be released into the human body and lead to implant failure [7,8]. For this reason, new aluminium and vanadium-free titanium alloys are being developed [9,10]. Based on commercially pure titanium grade 4, this new material is alloyed with elements which are either essential or for which no hazards are known. The developed alloys are required to meet the mechanical properties of the current standard alloy Ti Al6 V4 [10].

Surface modification methods may improve the tribological properties. Such techniques involve for instance processes like PVD/CVD [11,12], laser cladding [13], thermal spraying [14], hydrothermal treatment [15], sol-gel coating [16], or anodic oxidation [17,18]. One effective method providing plasma assisted electrochemical conversion of the metallic surface into a ceramic oxide layer is the plasma electrolytic oxidation (PEO) or micro arc oxidation (MAO) technique [19,20,21,22]. The PEO coating’s properties, e.g., morphology, porosity, composition, and thickness depend on the variation of the electrical plasma-electrolytic oxidation process parameters [22,23,24,25] as well as on the chemical composition of the chosen electrolytes [26,27,28,29]. Functional coatings in order to improve the mechanical properties [30,31,32], corrosion resistance [33,34], or biocompatibility [35,36,37] of titanium materials can be obtained.

In the present work, PEO coatings are developed on novel commercially pure titanium grade 4+ alloys investigating the influence of the electrical PEO process parameters on the wear and tribocorrosion behavior.

## 2. Materials and Methods

### 2.1. Sample Preparation

The substrate for the PEO treatment was a novel titanium alloy based on CP titanium grade 4+ material [10]. The elemental composition of the used alloy is shown in Table 1. It is alloyed with slightly enhanced oxygen and silicon contents compared to commercially pure titanium, resulting in beneficial mechanical properties. A cast rod of Ø 80 mm was sliced into plate shaped samples with dimensions of approx. 35 mm × 35 mm and 1 mm thickness. Prior to the PEO coating process, the samples were wet ground gradually using 180# to 1000# grit SiC abrasive paper. All specimens were cleaned with distilled water and degreased with ethanol in an ultrasonic bath for 10 min.

### 2.2. Plasma Electrolytic Oxidation

PEO treatments were carried out using a power unit Sorensen SGI 800/6 (San Diego, CA, USA). A two-electrode electrochemical cell setup was used with the titanium samples exposing a surface area of 23.9 cm${}^{2}$. A cylindrical platinized stainless steel mesh with a diameter of 10 cm served as the cathode. Plasma electrolytic oxidation experiments were carried out applying a regular two-level factorial design of experiments. Herein, current density, frequency and duty cycle were varied and its influence with respect to wear resistance is being investigated. Current density was varied from 50 mAcm${}^{-2}$ to 125 mAcm${}^{-2}$, repetition rate from 50 s${}^{-1}$ to 250 s${}^{-1}$ and duty cycle 50% to 80%. The PEO processing parameters were chosen based on suitable instrument specific key figures. In Table 2, the resulting DoE with the corresponding parameter variation is shown.

For the PEO treatment, an alkaline electrolyte was used based on NaOH and NaAlO${}_{2}$ in order to generate alumina rich coatings. The electrolyte composition is displayed in Table 3. PEO experiments were conducted under galvanostatic conditions setting a maximum voltage limit of 450 V. The accumulated charge densities were set constant at 210 Ccm${}^{-2}$ resulting into PEO processing times between 30 min and 70 min. During the PEO process, the electrolyte bath temperature was kept constant at 20 °C using a LAUDA water recirculation unit (Lauda-Königshofen, Germany). After the PEO process, the samples were cleaned with distilled water and air blasted.

### 2.3. Characterization

Investigation of the PEO coating’s morphology and cross-sectional microstructure was performed by scanning electron microscopy (SEM, Philips XL 40) equipped with an electron dispersive X-ray spectroscopy (EDS) detector. Cross sectioned samples were mounted with thermoplastic resin and progressively polished using an SiC paper followed by 1 μm diamond paste. Coating thickness measurements were carried out computerized on cross-sectional images at 100 different points and the average values were calculated. Porosity was determined by image evaluation of the SEM micrographs using ImageJ 1.53 software. The procedure involved thresholding of the original SEM micrographs yielding a binary image. The critical threshold was adjusted by the abrupt intensity onset in the grayscale histogram. Due to the large number of unidentifiable structures, a size limit of 0.5 μm was used to filter out these small structures. A minimum circularity of 50% was chosen.

The coating’s roughness was measured using a tactile profilometer MarSurf XR1 (Göttigen, Germany) at three different positions on each sample, and the average roughness values were determined.

Phase composition of the PEO coatings was analyzed by grazing incidence X-ray diffraction (GAXRD, Bruker D8 ADVANCE, Billerica, MA, USA) with an acceleration voltage of 40 kV and a beam current of 30 mA emitting Cu-K${}_{\alpha }$ radiation (λ = 1.54 Å). In order to reduce the contribution of the substrate, a Goebel mirror was used with a fixed angle of incidence of 7, a step size of 0.04, and a scan range from 10 to 100. After the scans, phase analysis was performed using Bruker DIFFRAC.EVA 6 software.

Wear experiments were carried out on PEO treated plate shaped samples immersed in simulated body fluid (Hank’s solution with addition of 0.1 M H${}_{2}$O${}_{2}$ in order to mimic a natural inflammation reaction induced by the surgical process of introducing a medical implant). The applied normal load, sliding velocity, and sliding distance was kept constant for all the measurements at 1 N, 5 mms${}^{-1}$ and 100 m, respectively. A ball on flat tribosystem was used with a 6 mm diameter alumina ball (supplier: TIS GmbH, Gauting, Germany; purity > 99% Al${}_{2}$O${}_{3}$, 1250–1700 HV) acting as the counter body. Due to abrasion of the counter body, a fresh ball was used for every experiment. The stroke length used in all experiments was 5 mm. Open circuit potential evolution was investigated in this study. The test protocol consisted of three phases: before sliding, during sliding and after sliding. At first, the plates were mounted into the tribocorrosion cell and OCP values were monitored for 20 h to attain stable potential values. After the initial stabilization, the tribological contact was established between the PEO treated plate shaped sample and the counter ball. After the sliding period, the contact has been removed. The evolution of OCP was continuously monitored by a PC controlled potentiostat throughout the whole tribocorrosion process. After the tribological tests, the wear track profile was acquired using a tactile profilometer determining average wear depth and wear width. Assuming a cylindrically shaped wear track, the wear volume *V* was calculated using the equation

(1) $$V\approx \frac{2}{3}dhsF^{-1}L^{-1},$$

where *d*, *h* and *s* are width, depth and length of the worn scar, *F* is the normal load (*F* = const. = 1 N) and *L* is the sliding distance being 100 m.

Finally, the test results were evaluated using the software DesignExpert^®^ 12, (Minneapolis, MN, USA) in order to analyze the influence of the PEO parameter settings on the coating’s tribological performance.

The mechanical properties of the PEO layers were investigated by nanoindentation measurements. A nanoindentation instrument (Anton Paar, Ostfildern, Germany) equipped with a Berkovich indenter was used. In addition, 36 indentations were applied on cross-sectioned samples in a 6 × 6 matrix, respectively. The maximum applied force was set constant to 20 mN, the intervall between the indents was set to 10 μm. The Oliver & Pharr method was used to evaluate the data applying a power law fit of the unloading curve (98% < F${}_{max}$ < 40%) [38].

In order to examine the PEO coatings’ barrier properties, electrochemical impedance spectroscopy (EIS) was carried out. The measurements were conducted with a potentiostat (ZAHNER Zennium, Kronach, Germany) with an excitation amplitude of ±10 mV in a frequency range from 10 mHz to 100 kHz. As for the wear experiments, a naturally aerated Hank’s solution was used..

## 3. Results

### 3.1. Wear Test

**Figure 1:**
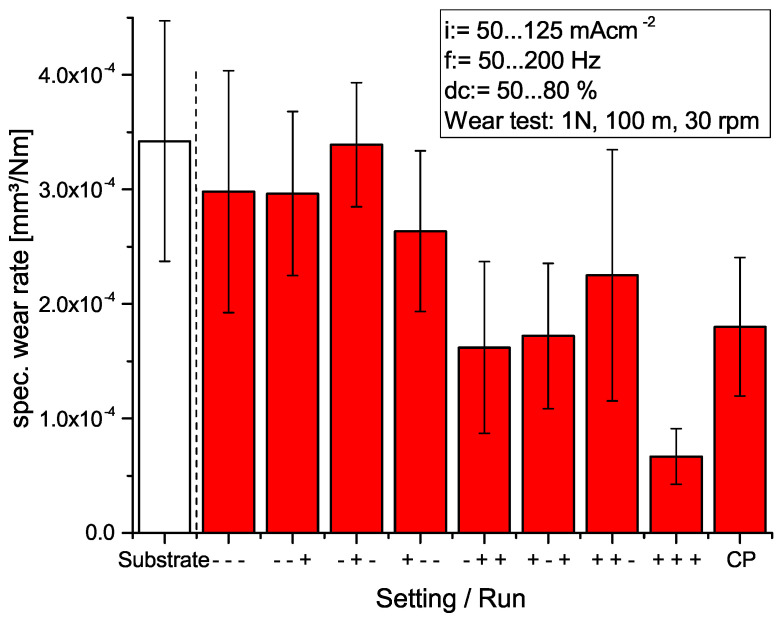
Obtained specific wear rates of the PEO coatings depending on the set PEO parameters. DoE notation is arranged in the following order: (current density/repetition rate/duty cycle).

**Figure 2:**
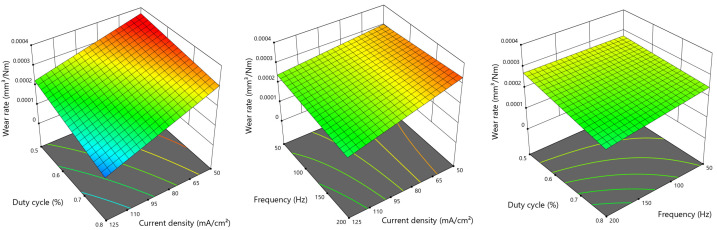
Influence of PEO parameters on the tribological behavior of the PEO coatings.

In Figure 1, the results of the wear tests for the PEO coatings obtained under the varied DoE conditions are summarized. Wear test parameters were set constant at a normal load of 1 N corresponding to a Hertzian pressure of approx. 550 MPa, a sliding distance of 100 m and a stroke frequency of 30 rpm in order to simulate a typical load scenario for a walking person. The counterpart was the specific wear rates that vary between maximum values of 3.5 × 10${}^{-4}$ mm${}^{3}$N${}^{-1}$m${}^{-1}$ for the setting (−+−) and minimal achieved wear of 7 × 10${}^{-5}$ mm${}^{3}$N${}^{-1}$m${}^{-1}$ for (+++) conditions. Compared to the substrate material, the wear is reduced by a factor of 4.3 for the setting (+++). However, for some PEO settings, no significant improvement could be obtained, e.g., (−−−) and (−−+), while, for (−+−) conditions, an even slightly worse wear behavior is observed. From the bar chart in Figure 1, it can be concluded that the wear performance strongly depends on the chosen PEO parameters; in particular, it improves for higher current densities and high duty cycles. The interaction with the frequency setting remains unclear, since, for high frequencies, both degradation (−+−) and improvement (+++) are obtained. In order to further investigate the PEO parameters’ interactions, a 2FI model was applied. The resulting graphs are presented in Figure 2. From the 3D model graphs, it can be concluded that, in fact, an increasing current density, frequency and duty cycle leads to improved wear resistance. Strongest interactions can be observed for dc↔i and f↔i, whereas dc↔f has a rather minor influence on the wear behavior.

**Figure 3:**
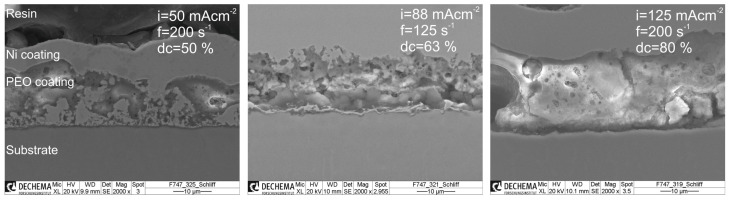
SEM micrographs of cross-sections of the generated PEO coatings for the DoE settings (−+−), (CP) and (+++).

In Figure 3, the SEM cross sectional micrographs of PEO coated samples assigned with the poorest wear behavior (−+−, left side) and the best wear behavior (+++, right side) are shown. The SEM micrograph in the middle belongs to the DoE’s center point sample. Obviously, the coating’s morphology changes with varying current density, frequency and duty cycle. In Table 4, the coating’s thickness, density and roughness depending on the PEO settings are summarized. It can be seen that the thickness remains relatively constant for all PEO settings ranging from 12–17 μm. This can be attributed to the specific charge density which was set constant to 210 Ccm${}^{-2}$ for all experiments.

**Figure 4:**
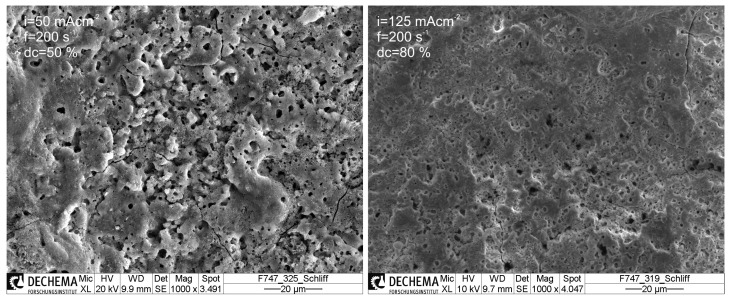
SEM micrographs of cross-sections of the generated PEO coatings for the DoE settings (−+−), (CP) and (+++).

In Figure 4, the scanning electron micrographs of the coating surfaces obtained under (−+−) and (+++) conditions are shown, respectively. The total applied charge density is for both samples 210 Ccm${}^{-2}$. Both coatings show a homogeneous morphology, whereby the pores are uniformly distributed over the surface. However, obviously the relief changes from a surface exhibiting voids and microcracks (−+−) to a more uniform and smooth topography (+++). Microscopic image evaluation reveals that the average pore diameter decreases with increasing current density and duty cycle, while the average pore density, i.e., the occurrence of microdischarges increases. This is accompanied with an increase of the overall porosity for the coatings obtained under low duty cycles (Table 4). Egorkin et al. attributed this behavior to an insufficient energy release during the PEO process which is not able to efficiently re-melt and solidify the already formed ceramic layer [39]. Consequently, they found that, for increasing frequency and duty cycle, the porosity decreases and the coating thickness increases.

The coating’s porosities vary between 4% for the (+++) and 25% for the (−−−) setting. In particular, the coatings generated at high repetition rates exhibit less and smaller pores, which has a positive effect on their wear performance, since the probability of the break out of abrasive coating particles is reduced.

Overall, the average roughness is increasing for increasing current densities, i.e., from R${}_{a}$ = 2.8 µm for (−−−) up to R${}_{a}$ = 4.6 µm for (+−−) configuration. Smoother surfaces can be obtained for higher frequencies, i.e., for the (−+−) setting, roughness is slightly decreasing to R${}_{a}$ = 2.6 µm, and a more significant effect can be observed comparing (+−+) and (+++) configuration with R${}_{a}$ = 5.0 µm and R${}_{a}$ = 3.5 µm, respectively.

Comparing the average roughness values R${}_{a}$ before and after tribotest, the results show that for all specimens the surface becomes smoother (0.1 μm < R${}_{a}$ < 0.5 μm), suggesting that, during the wear test, local surface peaks get abraded and homogeneous abrasion sets in.

However, from the SEM micrographs, it can be seen that obviously also the coating’s adhesion to the substrate is increasing as well with increasing current density and duty cycle. This plays a relevant role with respect to the wear behavior. As a result, from the experiments, the wear obtained for (−+−) conditions shows an even poorer behavior than the pristine substrate material, despite the fact that the coating showed a low average roughness. Most likely, this can be attributed to poor adhesion properties leading to an accumulation of abrasive particles in the wear track and hence an increased third body abrasion (Figure 3, left). Furthermore, the porosity is relatively high (17 ± 5%), favoring a rupture of ceramic coating particles. For (+++) conditions, a relatively thick, dense and smooth coating (thickness d = 17.5 ± 3.5 µm; porosity P = 4 ± 2%, R${}_{a}$ = 3.5 µm) with good adhesion could be generated, exhibiting the best wear properties within the conducted DoE.

Noteworthy abrasion could be observed on the alumina balls after tribocorrosion. Calculating the residual spherical volume of the alumina balls after the tests, the abrasion can be determined to be in the range of 1 × 10${}^{-6}$ mm${}^{3}$N${}^{-1}$m${}^{-1}$ to 5 × 10${}^{-7}$ mm${}^{3}$N${}^{-1}$m${}^{-1}$, depending on the mechanical properties of the PEO coating. It should be taken into account that severe abrasion due to third-body wear wear might also damage the ceramic ball [40].

### 3.2. Morphology and Phase Composition

**Figure 5:**
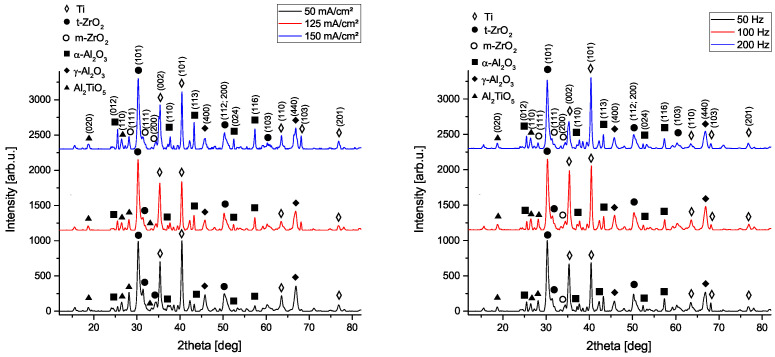
XRD patterns of PEO coatings obtained at different current densities (**left** side) and repetition rates (**right**). Residual process parameters were kept constant, respectively.

In Figure 5, the XRD patterns for PEO coatings generated under varying current density (left) and repetition rate (right) are shown. Duty cycle is kept constant at 80%, respectively. Evaluation of the XRD patterns reveals that, besides the titanium substrate, the formation of aluminium titanate (TiA${}_{2}$O${}_{5}$), α-alumina (corundum), γ-alumina and tetragonal zirconia can be observed as well. The amount of formed Al${}_{2}$O${}_{3}$ increases with increasing current density and frequency, where the amount of incorporated ZrO${}_{2}$ decreases slightly with increasing current density but is widely independent of the applied frequency.

In general, for high repetition rates and higher current densities, the generation of α-Al${}_{2}$O${}_{3}$ is favored, where the amount of TiAl${}_{2}$O${}_{5}$ decreases. This behavior can be attributed to the thermal instability of tialite above temperatures of 750 °C, leading to its decomposition into alumina and rutile [41]. Since the locally achieved temperatures increase with higher current densities and frequencies, the amount of formed corundum phase is approx. 12% for the (−−−) setting, where for (+++) up to 54% of generated crystalline phase consists of alumina. Moreover, not only the total amount of formed alumina but also the α:γ ratio is shifted to higher values for increasing repetition rates and current densities, in particular from approx. 1:3 (−−+) to 1:1 (+−+) and 2:1 (+++) configuration. This behavior can be attributed to the more efficient transformation of the metastable γ-alumina into the thermodynamically stable corundum phase due to higher final voltages obtained under high currents. The amount of incorporated zirconia remained more stable ranging between 11% and 26% for all generated coatings; however, as mentioned above, a reduction can be determined depending on the current density. It is noteworthy that the high temperature tetragonal phase is formed instead of the naturally occurring monoclinic phase, indicating high local temperatures during the PEO process (T > 2643 K). Due to grazing incidence measurement, the contribution of the titanium substrate to the XRD patterns is negligible. It should be noted that, under grazing incidence, the intensity of background scattering is enhanced, which was taken into account for quantification of the phases. However, quantification must be assumed to show a final resolution of ±5%.

It is well known that the formation of residual compressive stresses, e.g., in the use of shot peening further enhances the wear performance [42]. The high energy input during the PEO process induces high residual compressive stresses which beneath the inherent material properties of the formed phases improve the wear behavior of PEO-coated materials [43].

### 3.3. Nanoindentation Measurements

**Figure 6:**
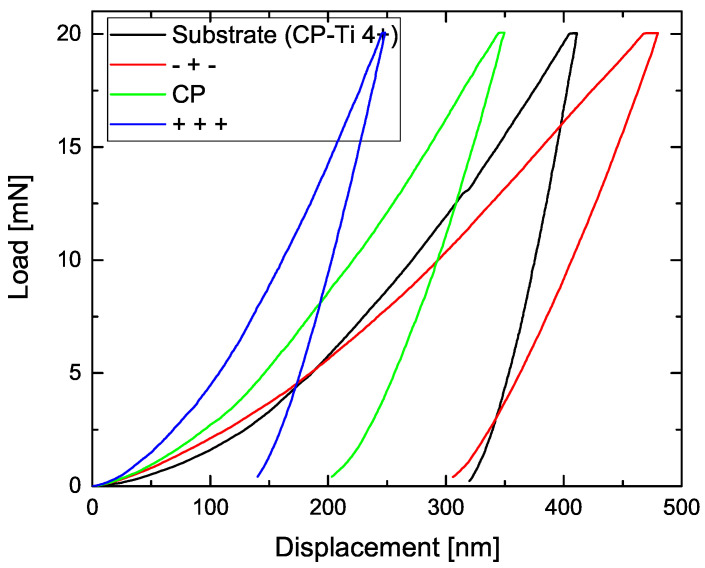
Load displacement curves for the pristine CP-Ti 4+ material and PEO coatings obtained under the DoE settings (−+−), (CP) and (+++).

Figure 6 represents the load displacement curves for the pristine material and three PEO coated samples representing the DoE modifications (−+−), (CP) and (+++), (for experimental details, refer to Table 2) under a constant load of 20 mN. The measurements were performed on polished cross sectional samples. From the curve’s gradient, the values of characteristic mechanical parameters like hardness and elasticity were calculated using the Oliver and Pharr method by applying a power law fit of the unloading curve [38]. From the development of the curves, it can be concluded that the PEO coatings are significantly harder than the pristine material; however, the applied electrical parameters show a huge influence on the coatings’ mechanical properties. In Table 5, the calculated values for relevant mechanical parameters, i.e., reduced Young’s modulus $\frac{1}{E^{*}}=\frac{1-\nu_{1}^{2}}{E_{1}}+\frac{1-\nu_{2}^{2}}{E_{2}}$, hardness *H* and Plasticity Index *P.I.* for the different DoE configurations are listed, respectively.

Comparing the results of the nanoindentation measurements with the DoE adjustments, it can be concluded that, for increasing current density and frequency, both hardness and H/E* ratio of the PEO coatings increase. In general, an increased H/E value is considered to have a beneficial influence on a material’s wear resistance [44]. On the other hand, with increasing hardness, the plasticity index decreases, since less work is converted into plastic deformation.

**Figure 7:**
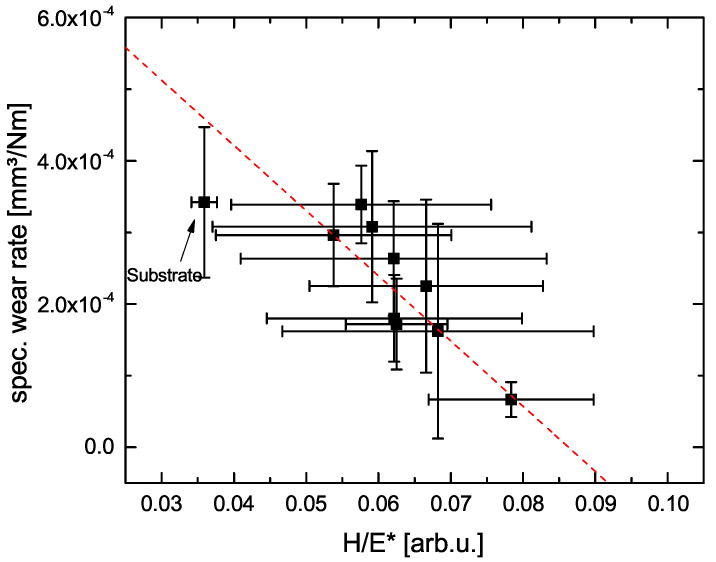
Specific wear rate as a function of the obtained ratio H/E*. The red dashed line is a guide to the eye.

**Figure 8:**
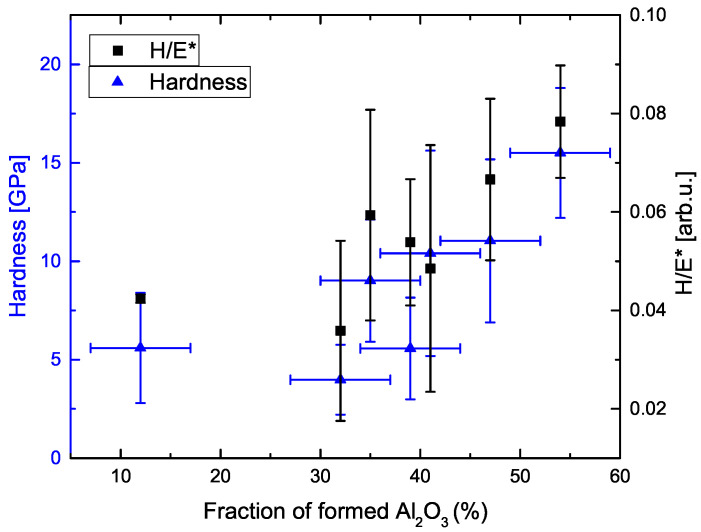
Hardness H and ratio H/E* depending on the amount of total formed alumina (α+γ phase).

In Figure 7, the wear rate of the obtained PEO coatings is plotted as a function of the ratio H/E*. It can be seen that, for increasing H/E*, the wear rate decreases. Comparing the achieved H/E* ratios, they are in the range between approx. 0.04 and 0.08 corresponding to values for hardened steel (H/E ≈ 0.02) and soda lime glass (H/E ≈ 0.08). Hence, it can be concluded that, depending on the PEO parameters, the wear behavior can be adjusted selectively. The reason for this behavior can be identified plotting the ratio H/E* as a function of the generated amount of alumina (Figure 8). It is obvious that, for an increasing fraction of formed alumina, H/E* is increasing as well, since the obtained coatings are substantially harder (Table 5).

### 3.4. Electrochemical Impedance Spectroscopy

**Figure 9:**
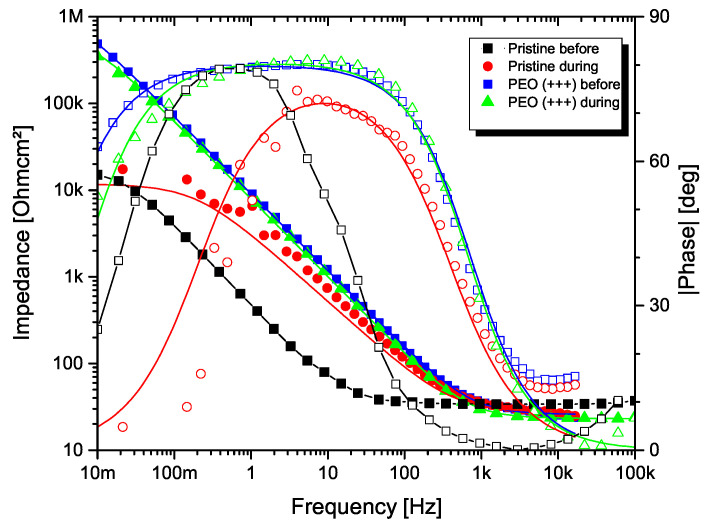
EIS spectra in Bode plot of the pristine titanium material and a PEO treated sample before and during a tribocorrosion test in Hank’s solution.

In Figure 9, the Bode plot of the electrochemical impedance spectra of the pristine titanium as well as a PEO treated sample (DoE configuration (+++)) are presented. Spectra were obtained in the range from 10${}^{-2}$ Hz to 10${}^{5}$ Hz. The plot shows the electrochemical impedance spectra before and during a tribocorosion test in Hank’s solution with the addition of 0.1 M H${}_{2}$O${}_{2}$. The lines show a fit of the obtained data according to a simple Randles cell, i.e., R${}_{el}$(R${}_{i}$CPE${}_{i}$) for the pristine material and R${}_{el}$(CPE${}_{p}$(R${}_{p}$(R${}_{i}$CPE${}_{i}$))) for the PEO treated sample. The used model circuits describe the behavior for a passive material with a rather compact barrier layer immersed in a corrosive electrolyte [45] and those of a coating consisting of an outer porous structure and an inner dense barrier layer [46].

It can be observed that, under friction, size and shape of the Bode diagram change. During the wear process, the impedance decreases for pristine and PEO coated material. For the PEO treated sample, the impedance measured at 10${}^{-2}$ Hz decreases from 490 kΩcm${}^{2}$ to 373 kΩcm${}^{2}$, indicating that the polarisation resistance of the PEO coating is less effective due to wear debris. However, the coating is still present as no reduction of the impedance towards the value of the pristine material takes place. Moreover, the phase shift remains comparatively stable, indicating no significant change of the capacitive behavior, i.e., coating failure.

In case of the pristine material, a capacitance shift towards the high frequency region during the tribocorrosion test can be observed, indicating an increase in the transfer resistance. This behavior can be interpreted by a decrease in the repassivation ability of the surface, i.e., a failing of the thin titania passive layer [47].

## 4. Conclusions

Plasma electrolytic oxidation on CP titanium grade 4+ materials exhibits significantly improved wear properties compared to the base material. The main effects for the generation of wear-resistant coatings in terms of the varied parameters can be identified as the current density and repetition rate. GAXRD phase analysis shows that, in addition to the titanium substrate, the formation of aluminium titanate (TiAl${}_{2}$O${}_{5}$), tetragonal zirconia (t-ZrO${}_{2}$) as well as corundum (α-Al${}_{2}$O${}_{3}$) can be observed. This contributes to an increase in the hardness of the generated PEO coatings up to 15.5 GPa (H/E* ≈ 0.08) and an improved wear resistance of 6.7 × 10${}^{-5}$ mm${}^{3}$ N${}^{-1}$m${}^{-1}$ vs. 3 × 10${}^{-4}$ mm${}^{3}$ N${}^{-1}$m${}^{-1}$ compared to the pristine material, respectively. Depending on the electrical parameters, the phase fraction and the layer morphology can be varied in a wide range. The amount of formed alumina increases with increasing current density and frequency. Likewise, thicker and denser layers are obtained by increasing the current density and the duty cycle, while an increase of the repletion rate leads to the formation of smoother coatings. Nanoindentation measurements show that the wear rate depends on the H/E* ratio, i.e., the total wear decreases with increasing plasticity index, which is due to the increased formation of alumina phase and the optimized coatings morphology.

## Figures and Tables

**Table 1:** Elemental composition of the titanium substrate.

Element	Ti	O	Fe	C	Si
Composition [wt.%]	Bal.	0.44	0.50	0.08	0.50

**Table 2:** Design of experiments for the PEO process.

Configuration	Current Density *i*	Frequency *f*	Duty Cycle *dc*
*i/f/dc*	[mAcm${}^{-2}$]	[Hz]	[%]
−−−	50	50	50
−−+	50	50	80
−+−	50	250	50
+−−	125	50	50
−++	50	250	80
+−+	125	50	80
++−	125	250	50
+++	125	250	80
CP	88	125	63

**Table 3:** Electrolyte composition for the PEO process.

Substance	Concentration	pH	Conductivity
	[gL${}^{-1}$]		[mScm${}^{-1}$]
NaOH	2		
NaAlO${}_{2}$	40	13	47
m-ZrO${}_{2}$	4		

**Table 4:** Thickness, roughness and cross-sectional porosity of the generated PEO coatings.

DoE Setting	Coating Thickness	av. Roughness R${}_{a}$	Porosity
[*i/f/dc*]	[μm]	[μm]	%
−−−	14.5 ± 3.6	2.8	24.6 ± 8.7
−−+	15.5 ± 4.4	2.7	11.3 ± 1.2
−+−	15.5 ± 4.0	2.6	16.7 ± 4.8
+−−	15.0 ± 4.9	4.6	14.1 ± 1.0
−++	15.3 ± 3.3	3.5	14.6 ± 3.2
+−+	12.1 ± 3.6	5.0	6.5 ± 1.9
++−	13.2 ± 2.5	4.8	7.0 ± 2.9
+++	17.5 ± 3.5	3.5	4.0 ± 2.3
CP	15.2 ± 4.7	4.1	9.1 ± 2.8

**Table 5:** Obtained mechanical properties of the PEO coatings for the given experimental configurations derived from load displacement curves as shown in Figure 6.

DoE Setting	Hardness	Red. Young’s Modulus	Plasticity Index
[*i/f/dc*]	[GPa]	[GPa]	arb.u.
Substrate	4.5 ± 0.2	129 ± 9	
−−−	5.8 ± 2.6	101 ± 67	0.62 ± 0.31
−−+	5.9 ± 2.9	110 ± 22	0.62 ± 0.08
−+−	4.0 ± 2.8	105 ± 37	0.48 ± 0.09
+−−	9.0 ± 3.1	145 ± 26	0.61 ± 0.13
−++	10.4 ± 5.2	152 ± 30	0.63 ± 0.15
+−+	9.5 ± 2.9	155 ± 39	0.62 ± 0.08
++−	11.0 ± 4.1	166 ± 43	0.57 ± 0.10
+++	15.5 ± 3.3	198 ± 28	0.78 ± 0.03
CP	10.2 ± 3.2	168 ± 29	0.59 ± 0.14

## Data Availability

Not applicable.
